# Efficacy and safety of immune checkpoint inhibitors plus recombinant human endostatin therapy as second-line treatment in advanced non-small-cell lung cancer with negative driver gene: a pilot study

**DOI:** 10.3389/fonc.2023.1210267

**Published:** 2023-11-07

**Authors:** Bo Yang, Yuzhi Li, Jie Deng, Hui Yang, Xiang Sun

**Affiliations:** Department of Oncology, The Third Affiliated Hospital of Anhui Medical University (The First People’s Hospital of Hefei), Hefei, Anhui, China

**Keywords:** non-small cell lung cancer, recombinant human endostatin, anti-angiogenesis, immune checkpoint inhibitors, second-line

## Abstract

**Background:**

Immune checkpoint inhibitors (ICIs) have become the standard second-line treatment for advanced non-small cell lung cancer (NSCLC). Recent findings indicating an intertwined regulation of vascular endothelial growth factor (VEGF) signaling and immunosuppression in the tumor microenvironment suggest that the combination of ICIs and angiogenesis inhibitors could have synergistic antitumor activity, along with favorable tolerability. However, ICIs plus anti-angiogenesis therapy has not been widely evaluated. The purpose of this pilot study was to evaluate the efficacy and safety of ICIs plus recombinant human (rh)-endostatin as second-line treatment in advanced NSCLC with negative driver gene.

**Method:**

Prospectively evaluated the efficacy and safety of ICIs plus rh-endostain as second-line treatment in advanced NSCLC with negative driver gene. The primary endpoints of the study were progression-free survival (PFS) and overall survival (OS). The secondary endpoints were objective response rate (ORR), disease control rate (ORR), and safety.

**Results:**

A total of 34 patients were recruited in this study. 18 patients received ICIs plus anti-angiogenesis therapy (ICIs combination therapy), and 16 patients received ICIs monotherapy. DCR was 88.9% vs 43.8% (*P* = 0.009). Median PFS (mPFS) was 8.3 months vs. 3.7 months (HR = 0.276, 95% CI 0.125-0.607, *P* = 0.001). Median OS (mOS) was 18.0 months vs 9.6 months (HR=0.364, 95% CI 0.147-0.902, *P*=0.009). In multivariate Cox regression analysis, ICI combination therapy prolonged PFS (HR = 0.069, 95% CI 0.019-0.185, *P* < 0.001) and OS (HR = 0.044, 95% CI 0.011-0.185, *P* < 0.001). We did not observe a significant difference in the incidence of adverse events (AEs) between the two groups (P > 0.05).

**Conclusions:**

Compared with ICIs monotherapy, ICIs combination therapy improves clinical response in patients with advanced NSCLC with negative driver gene, significantly prolongs PFS and OS, and does not significantly difference the incidence of AEs.

## Introduction

According to the Global Cancer Statistics Report 2020, primary lung cancer is the second most common malignant tumor in the world, following breast cancer, and it is the leading cause of cancer-related death (1). The incidence and mortality are 11.4% and 18.0%, respectively (1). Non-small-cell lung cancer (NSCLC) is the most common type of lung cancer, accounting for approximately 85% of lung cancer, with a 5-year survival rate of only 16% (2). Unfortunately, most patients have been in the advanced stages when clearly diagnosed and have lost the opportunity for surgery, leading to a poor prognosis (3, 4).

In recent years, with the growing understanding of tumor immune escape and molecular biology, along with the development precision therapy for tumor. Immune checkpoint inhibitors (ICIs) have attracted much attention. Particularly, the emergence of Programmed cell Death-1 (PD-1) and Programmed cell Death-Ligand 1 (PD-L1) inhibitors has brought breakthrough treatment progress for patients with advanced NSCLC, increasing the five-year survival rate from 4.9% to 16%, significantly improving the prognosis of patients (5). ICIs have become an indispensable therapeutic approach for advanced NSLCL. IMpower130 study (6) suggested that atezolizumab plus chemotherapy in first-line treatment of advanced NSCLC patients without EGFR and ALK mutations significantly prolonged median progression-free survival (mPFS) (7.0 months vs 5.5 months, HR = 0.64, *P* < 0.0001) and median overall survival (mOS) (18.6 months vs 13.9months, HR = 0.79, *P* = 0.033), compared with chemotherapy monotherapy. KEYNOTE-407 trial (7) revealed that pembrolizumab plus chemotherapy significantly improved mPFS (6.4 months vs 4.8 months, HR = 0.56, *P* < 0.001) and mOS (15.9 months vs 11.3 months, HR = 0.64, *P* < 0.001) in patients with advanced NSCLC, and did not significantly increase the incidence of adverse events (AEs). ORIENT-12 study (8) also demonstrated that sintilimab plus chemotherapy significantly prolonged mPFS (5.5 months vs 4.9 months, *P* < 0.00001) in advanced NSCLC and had a tendency to benefit from OS (HR = 0.567, *P* = 0.01701). These findings suggest that ICIs combination therapy has a significant effect in the first-line treatment of advanced NSCLC.

Although ICIs monotherapy has become the new standard of second-line treatment for advanced NSCLC (including squamous cell carcinoma and adenocarcinoma), the objective response rate (ORR) remains low, with only a few patients experiencing long-term survival benefits (9, 10). Furthermore, due to the primary resistance of some patients to ICIs and the acquired resistance of some patients after treatment, the benefit groups of ICIs monotherapy also have great limitations (11). Combination therapy could be one of the solutions to increase the fraction of responding patients to ICIs.

Over the past few decades, substantial evidence has demonstrated that the crucial role of tumor neovascularization in the growth, proliferation and metastasis of various solid tumors. Angiogenesis inhibitors can effectively degrade existing tumor vessels and suppress tumor neovascularization, improving the infiltration of immune cells in the tumor microenvironment, relieving the immunosuppressive state, and positively regulating the immune function (12, 13). In addition, ICIs can also enhance the efficacy of angiogenesis inhibitors by promoting vascular changes (13–15). Therefore, ICIs plus anti-angiogenesis therapy has synergistic antitumor effect and is a promising therapeutic regimen. The purpose of this pilot study was to evaluate the efficacy and safety of ICIs plus anti-angiogenesis therapy as a second-line treatment in advanced NSCLC with negative driver gene.

## Methods

### Patient selection

The inclusion criteria were as follows: (I) range in age from 18 to 80 years; (II) Eastern Cooperative Oncology Group performance status (ECOG PS)score of 0-2; (III) advanced NSCLC with first-line treatment failed; (IV) without driver gene mutation; (V) at least one target lesion could be evaluated according to Response Evaluation Criteria In Solid Tumors (RECIST, version 1.1); (VI) predicted survival time of more than three months; (VII) adequate organ function; (VIII) normal Cardiac function and electrocardiograph (ECG); (IX) signed informed consent form.

The exclusion criteria were as follows: (I) pregnant women, as well as men and women of reproductive age who were refusing to use appropriate contraception; (II) patients with a history of severe heart disease; (III) patients with severe allergy to ICI or rh-endostatin; (IV) patients suffering from active bleeding or at danger of bleeding; (V) leukocytes < 2 × 10^9^/L, neutrophils < 1 × 10^9^/L, or platelets < 50 × 10^9^/L.

### Study design and treatment

This pilot study was approved by the Ethics Committee of the Third Affiliated Hospital of Anhui Medical University. A total of 34 patients with advanced NSCLC diagnosed by pathology or cytology in the Third Affiliated Hospital of Anhui Medical University, Chaohu Hospital of Anhui Medical University and Anhui Chest Hospital from March 2021 to March 2023 were collected. Non-randomized controlled study method was used in our study, and all patients and their families chose the treatment voluntarily. 18 patients received ICIs combination therapy, and 16 patients received ICIs monotherapy. ICIs including camrelizumab (200mg/3w, i.v. drip), sintilimab (200mg/3w, i.v. drip), pembrolizumab (200mg/3w, i.v. drip), tislelizumab (200mg/3w, i.v. drip). Anti-angiogenesis agent was recombinant human (rh)-endostatin (15mg/2.4 × 10^5^U/3ml/dose), which was given at a dose of 210 mg continuous intravenous pumping for 72 hours with an infusion pump every 3 weeks. Treatment continues until disease progression, unacceptable AEs occur, or the patient withdraws consent. Treatment response was assessed every 2 cycles of therapy according to RECIST (version 1.1), AEs was evaluated every cycle. All patients signed informed consent.

### Date collection and study objectives

The clinical data of all patients were recorded, including age, sex, smoking history, ECOG PS, surgical history, pathological type, tumor stage, metastatic site, first-line treatment regimen, and ICIs. The primary endpoints of this study were PFS and OS. The secondary endpoints were ORR, disease control rate (DCR) and safety. Tumor response was evaluated according to RECIST (version 1.1). AEs were assessed according to the Common Terminology Criteria for Adverse Events, version 5.0 (CTCAE 5.0).

### Statistical analysis

In this study, the statistical analyses were conducted using SPSS statistical software (version 26.0, SPSS, IBM Corporation, Armonk, NY, USA), and the data were presented as counts and percentages (%). Categorical variables were compared using Pearson X2 test or Fisher exact test. The survival curve was plotted using Kaplan-Meier method and Log-Rank test is used to compare survival date. Cox proportional hazard regression models was used for univariate and multivariate analysis. In this study, a two-sided hypothesis test was adopted, with *P<*0.05 was considered statistically significant.

## Results

### Baseline patient characteristics

**Figure 1:**
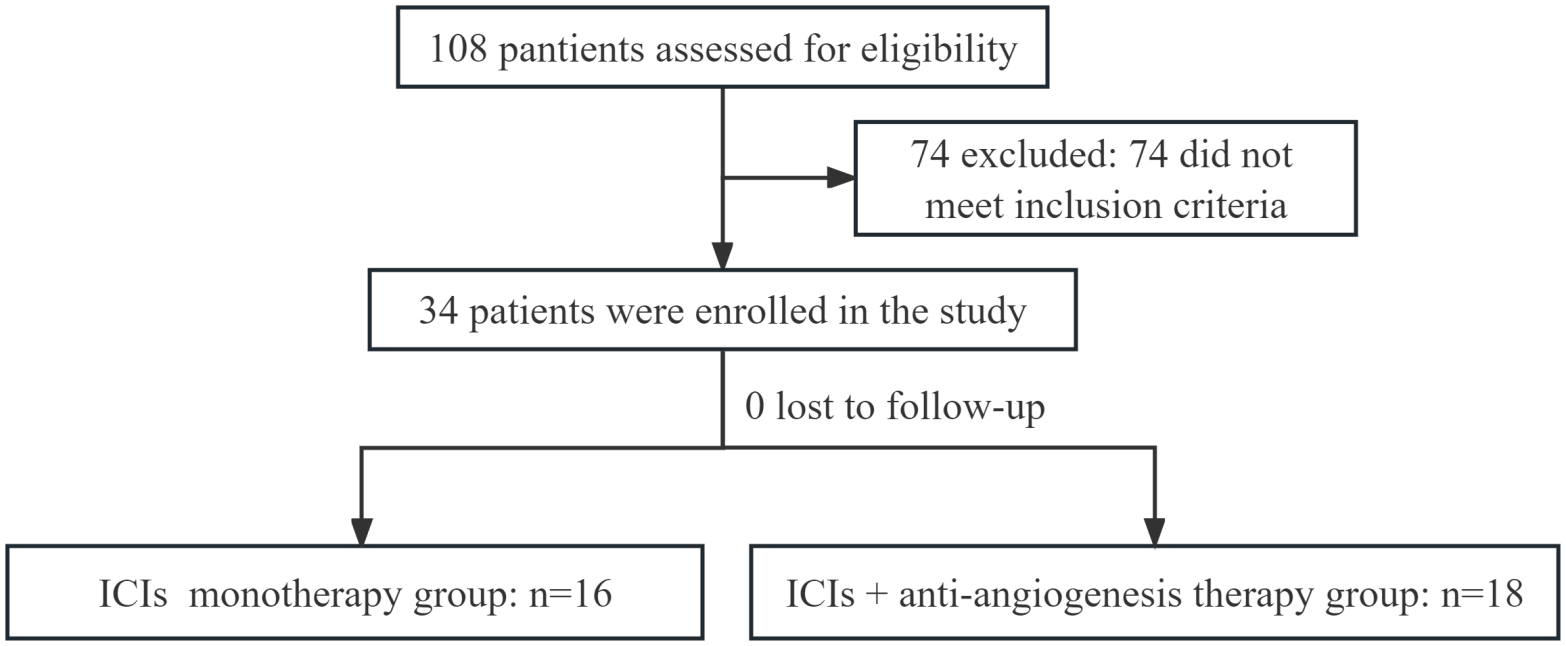
Diagram of the study.

A total of 34 patients were included in this study, and divided into ICIs combination therapy group and ICIs monotherapy group (**Figure 1**). The baseline clinical features of enrolled patients were listed in **Table 1**. All patients had failed first-line chemotherapy and without immunotherapy, and first-line treatment regimens were supplemented in **Table 1**. 18 patients were treated with ICIs combination therapy, including 13 patients of adenocarcinoma (72.2%), 10 patients of males (55.6%), 14 patients of ECOG PS ≤ 1, and the median age was 66.5 (range 52-79) years. 16 patients were treated with ICIs monotherapy, including 11 patients of adenocarcinoma (68.8%), 9 patients of males (56.3%), and 12 patients of ECOG ≤ 1, and the median age was 67.5 (range 54-80) years. In this study, there were four ICI agents: camrelizumab, sintilimab, pembrolizumab, and tislelizumab (**Figure 1**). There was no significant difference in baseline features between the two groups (P > 0.05). The last follow-up was on March 15, 2023.

**Table 1:** Baseline clinicopathological characteristics of patients.

Characteristics	ICI + anti-angiogenesis therapy	ICI monotherapy therapy	*P*
**Total case, n (%)**	18 (100.0)	16 (100.0)	
**Age(years)**			0.929
Media	66.5	67.5	
range	52-79	54-80	
**Gender, n (%)**			0.738
Male	10 (55.6)	10 (62.5)	
Female	8 (44.4)	6 (37.5)	
**Histology, n (%)**			1.000
Adenocarcinoma	13 (72.8)	11 (68.8)	
Squamous	5 (27.8)	5 (31.3)	
**ECOG performance status, n (%)**			1.000
0-1	14 (77.8)	12 (75.0)	
2	4 (22.2)	4 (25)	
**Smoking histology, n (%)**			0.732
Ever	8 (44.4)	9 (56.3)	
Never	10 (55.6)	7 (43.8)	
**TNM stage, n (%)**			
IV	18 (100.0)	16 (100.0)	
**First-line treatment regimen, n (%)** Pemetrexed plus platinum Paclitaxel plus platinum Gemcitabine plus platinum	7 (38.9) 8 (44.4) 3 (16.7)	6 (37.5) 8 (50.0) 2 (12.5)	0.923
Metastatic sites, n (%)			
Brain	4 (22.2)	3 (18.8)	1.000
Lung	7 (38.9)	7 (43.8)	0.475
Liver	3 (16.7)	4 (25.0)	0.703
Bone	6 (33.3)	6 (37.5)	1.000
Pleura	2 (11.1)	2 (12.5)	0.604
**ICIs agent, n (%)**			0.979
Camrelizumab	7 (38.9)	7 (43.8)	
Sintilimab	5 (27.8)	4 (25.0)	
Pembrolizumab	3 (16.7)	3 (18.8)	
Tislelizumab	3 (16.7)	2 (12.5)	

ECOG, Eastern Cooperative Oncology Group; ICIs, immune checkpoint inhibitors.

### Clinical efficacy

**Figure 2:**
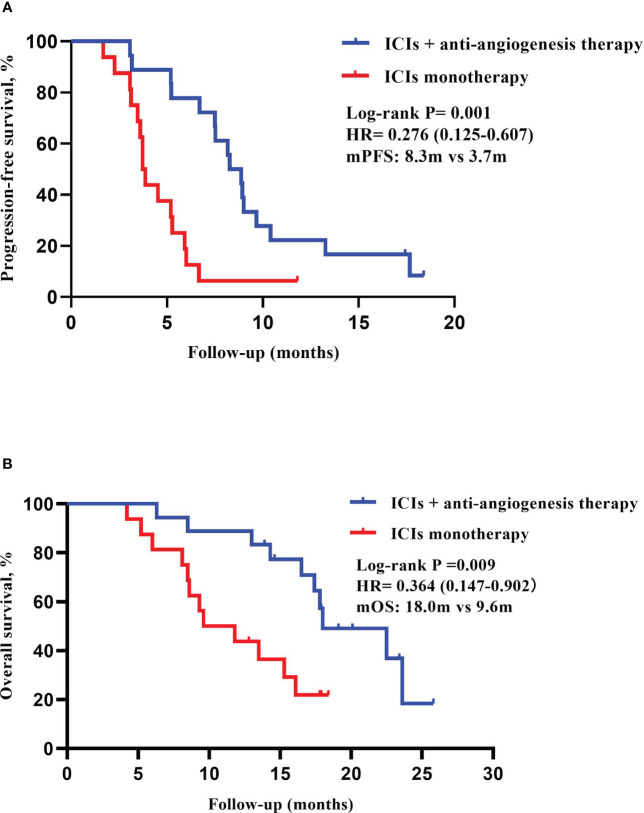
Kaplan-Meier curves of PFS and OS in ICIs plus anti-angiogenesis group (n=18) and ICIs monotherapy group (n=16). **(A)** Median progression-free survival (mPFS): 8.3 months (95% CI 6.811-9.722) vs 3.7 months (95% CI 3.385-4.082). **(B)** Median overall survival (mOS): 18.0months (95% CI 12.143-23.923) vs 9.6 months (95% CI 4.733-14.533).

Overall, no complete response (CR) patients were found in the two groups. The number of patients who got partial disease (PR), stable response (SD) and progressive disease (PD) were 4 patients (22.2%), 12 patients (66.7%) and 2 patients (11.1%) in the ICIs combination therapy group. Meanwhile, the number of patients who got PR, SD and PD were 1 patient (6.3%), 6 patients (37.5%) and 9 patients (56.3%) in the ICIs monotherapy group. The ORR was 22.2% vs. 6.3% (*P* = 0.34) and the DCR was 88.9% vs.43.8% (*P* = 0.009) (**Table 2**). Survival analysis showed that the mPFS in ICIs combination group (8.3 months, 95% CI 6.811-9.722) was higher than ICIs monotherapy group (3.7 months, 95% CI 3.385-4.082) (HR = 0.276, 95% CI 0.125-0.607, *P* = 0.001). At the same time, mOS (18.0 months, 95% CI 12.143-23.923) in the ICIs combination therapy group was higher than ICIs monotherapy group (9.6 months, 95% CI 4.733-14.533) (HR = 0.364, 95% CI 0.147-0.902, *P* = 0.009) (**Figure 2**).

**Table 2:** Treatment response assessed per RECIST version 1.1.

	ICIs + anti-angiogenesis therapy	ICIs monotherapy	*P*
Best overall response, *n* (%)			
CR	0	0	
PR	4 (22.2)	1 (6.3)	
SD	12 (66.7)	6 (37.5)	
PD	2 (11.1)	9 (56.3)	
ORR	4 (22.2)	1 (6.3)	0.340
DCR	16 (88.9)	7 (43.8)	0.009

ICIs, immune checkpoint inhibitors; CR, complete response; PR, partial response; SD, stable disease; PD, progression disease; ORR, objective response rate; DCR, disease control rate.

In univariate analysis of PFS, baseline age < 65 years (HR = 0.237, 95% CI, 0.094–0.602, *P* = 0.002), ECOG PS 0-1 (HR = 0.231, 95% CI 0.096-0.557, *P* = 0.001), no brain metastasis (HR = 7.847, 95% CI 2.781–22.138, *P* < 0.001), ICIs combination therapy (HR = 0.276, 95% CI 0.125–0.607, *P* = 0.001) had higher PFS. In multivariate cox models including baseline age < 65 years, ECOG 0-1, no brain metastases and ICIs combination therapy, baseline age < 65 years (HR = 0.130, 95% CI 0.039-0.435, *P* = 0.001), ECOG PS 0-1 (HR = 0.238, 95% CI 0.084-0.677, *P* = 0.007), no brain metastasis (HR = 12.998, 95% CI 3.339-50.602, *P* < 0.001), ICIs combination therapy (HR = 0.069, 95% CI 0.019-0.185, *P* = 0.001) was still significantly associated with increased PFS (**Table 3**). In the univariate analysis of OS, baseline age was < 65 years (HR = 0.149, 95% CI 0.044-0.511, *P* = 0.002), ECOG PS 0-1 (HR = 0.142, 95% CI 0.048-0.421, *P* < 0.001), no brain metastasis (HR = 5.774, 95% CI 2.225-14.982, *P* < 0.001), ICIs combination therapy (HR=0.268, 95% CI 0.108-0.667, *P* = 0.005) with higher OS. In multivariate cox models including baseline age < 65 years, ECOG PS 0-1, no brain metastases and ICIs combination therapy, age < 65 years (HR = 0.100, 95% CI 0.022-0.444, *P* = 0.002), ECOG PS 0-1 (HR = 0.151, 95% CI 0.042-0.543, *P* = 0.007), no brain metastasis (HR = 10.342, 95% CI 2.482-43.105, *P* = 0.001), ICIs combination therapy (HR=0.044, 95% CI 0.011-0.185, *P*=0.000) was still significantly associated with increased OS (**Table 4**).

**Table 3:** Univariate and multivariate analysis of progression-free survival.

Factors	Progression-free survival					
	Univariate analysis			Multivariate analysis		
	HR	95% CI	Log-rank *P*	HR	95% CI	Log-rank *P*
Age						
<65 vs ≥65	0.237	0.094-0.602	0.002	0.130	0.039-0.435	0.001
Gender						
Male vs female	1.129	0.551-2.315	0.740			
ECOG performance status						
0-1 vs 2	0.231	0.096-0.557	0.001	0.238	0.084-0.677	0.007
Smoking status						
Ever vs never	1.531	0.743-3.151	0.248			
Histology						
Adenocarcinoma vs squamous	0.507	0.222-1.155	0.106			
Surgery						
Yes or no	0.582	0.246-1.378	0.218			
Metastatic						
Brain						
Yes or no	7.847	2.781-22.138	< 0.001	12.998	3.339-50.602	< 0.001
Lung						
Yes or no	0.946	0.458-1.958	0.882			
Liver						
Yes or no	1.544	0.696-3.423	0.285			
Bone						
Yes or no	1.028	0.500-2.114	0.940			
Pleura						
Yes or no	2.373	0.792-7.109	0.123			
ICIs agent						
Camrelizumab vs sintilimab vs pembrolizumab vs tislelizumab	0.987	0.705-1.381	0.940			
Treatment therapy						
ICIs plus anti-angiogenesisi vs ICIs monotherapy	0.276	0.125-0.607	0.001	0.069	0.019-0.185	< 0.001

ECOG, Eastern Cooperative Oncology Group; ICIs, immune checkpoint inhibitors.

**Table 4:** Univariate and multivariate analysis of overall survival.

Factors	Overall survival					
	Univariate analysis			Multivariate analysis		
	HR	95% CI	Log-rank *P*	HR	95% CI	Log-rank *P*
Age						
<65 vs ≥65	0.149	0.044-03511	0.002	0.100	0.022-0.444	0.002
Gender						
Male vs female	1.377	0.587-3.232	0.463			
ECOG performance status						
vs 2	0.142	0.048-0.421	< 0.001	0.151	0.042-0.543	0.004
Smoking status						
Ever vs never	1.234	0.541-2.815	0.617			
Histology						
Adenocarcinoma vs squamous	0.768	0.323-1.825	0.550			
Surgery						
Yes or no	0.981	0.383-2.510	0.968			
Metastatic						
Brain						
Yes or no	5.774	2.225-14.982	< 0.001	10.342	2.482-43.105	0.001
Lung						
Yes or no	1.743	0.667-4.552	0.257			
Liver						
Yes or no	0.996	0.388-2.588	0.994			
Bone						
Yes or no	0.522	0.522-2.770	0.665			
Pleura						
Yes or no	2.560	0.700-9.360	0.155			
ICI agent						
Camrelizumab vs Sintilimab vs	0.927	0.638-1.347	0.691			
Pembrolizumab vs Tislelizumab						
Treatment therapy						
ICI plus anti-angiogenesis vs ICI monotherapy	0.268	0.108-0.667	0.005	0.044	0.011-0.185	< 0.001

ECOG, Eastern Cooperative Oncology Group; ICIs, immune checkpoint inhibitors.

**Table 5** lists the AEs, and there was no statistically significant difference between the two groups (P > 0.05). The incidence of grade 1-2 AEs was 72.2% in ICIs combination therapy group and 68.2% in ICIs monotherapy group. Grade 3-4 AEs in ICIs combination therapy group were leukopenia (5.5%), anemia (5.5%), hypertension (5.5%), hepatic dysfunction (5.5%), and pneumonia (5.5%). Grade 3-4 AEs in ICIs monotherapy group were anemia (6.3%), hypertension (6.3%), rash (6.3%), and diarrhea (6.3%). Additionally, no significant increase in the incidence or severity of toxicity was observed with rh-endostatin, nor was there a definite incidence of cardiotoxicity. The above AEs could be managed with symptomatic treatment, and no death caused by toxic and side effects occurred.

**Table 5:** Adverse events [*n* (%)].

Event	ICIs + anti-angiogenesis therapy		ICIs monotherapy		
	Grade1/2	Grade3/4	Grade1/2	Gade3/4	*P*
Any	13 (72.2)	4 (22.2)	11 (68.8)	3 (18.8)	0.591
Leucopenia	3 (16.7)	1 (5.6)	3 (18.8)		1.000
Anemia	7 (38.9)	1 (5.6)	5 (31.3)	1 (6.3)	0.738
Thrombocytopenia	1 (5.6)		0		1.000
Anorexia	7 (5.6)		5 (31.3)		0.729
Fatigue	5 (27.8)		4 (25.0)		1.000
Rash	3 (16.7)		2 (12.5)	1 (6.3)	1.000
Pruritus	3 (16.7)		2 (12.5)		1.000
Fever	2 (11.1)		3 (18.8)		0.648
Nausea/Vomiting	3 (16.7)		2 (12.5)		1.000
Diarrhea	4 (22.2)		2 (12.5)	1 (6.3)	1.000
Hypertension	3 (16.7)	1 (5.6)	1 (6.3)	1 (6.3)	0.660
Proteinuria	3 (16.7)		1 (6.3)		0.604
Hepatic dysfunction	3 (16.7)	1 (5.6)	3 (18.8)		1.000
Creatinine elevation	1 (5.6)		0		1.000
Pneumonia	1 (5.6)	1 (5.6)	1 (6.3)		1.000
Myalgia	3 (16.7)		1 (6.3)		0.604
Arthralgia	1 (5.6)		0		1.000
Hemoptysis/EpistaxIs	2 (11.1)		1 (6.3)		1.000
Adrenal insufficiency	1 (5.6)		0		1.000
Hypothyroidism	4 (22.2)		2 (12.5)		0.660
RCCEP	4 (22.2)		3 (18.8)		1.000

ICIs, immune checkpoint inhibitors; RCCEP, Reactive cutaneous capillary endothelial proliferation.

## Discussion

At present, lung cancer remains one of the most prevalent and deadliest malignant tumors worldwide. Among them, NSCLC, as the predominant type of lung cancer, accounts for approximately 85%, with the 5-year survival rate is only 16% (2). In recent years, immunotherapy represented by ICIs has made significant advancements in the field of tumor therapy. In particular, the development of PD-1 and PD-L1 inhibitors provides new treatment options for patients with advanced NSCLC, effectively treating squamous and non-squamous NSCLC and greatly improving the survival prognosis. Currently, ICIs are considered the standard second-line treatment for advanced NSCLC following failure of first-line treatment. However, the mPFS benefits from ICI monotherapy has been reported to be only in the range of 2.3 to 3.7 months (16, 17). Additionally, patients receiving ICIs monotherapy have many problems such as drug resistance and low remission rate, which can only benefit a small number of patients and fail to meet the existing clinical needs and our expectation (10, 11, 18, 19). Studies have demonstrated that angiogenesis inhibitors and ICIs can modulate tumor microenvironment and have potential synergistic mechanism, the combined application provides a promising prospect for antitumor therapy (13, 15, 20).

Angiogenesis is essential for growth, invasion, and metastasis of solid tumor. In 1971, Folkman (21) discovered tumor angiogenesis factors and proposed a new anti-angiogenesis approach for tumor treatment. Many studies have identified vascular endothelial growth factor (VEGF) as a key regulatory factor promoting tumor angiogenesis, and VEGF and VEGF receptor (VEGFR) signal transduction pathway is one of the most important pathways promoting angiogenesis (13, 22). Angiogenesis inhibitors mainly targets VEGF and VEGFR, and reverses the immunosuppressive state of tumor microenvironment by blocking signal transduction pathways or reducing VEGF expression level of tumor cells. Finally, angiogenesis inhibitors restrains tumor growth and metastasis by inhibiting the formation of tumor neovascularization, reducing or blocking the nutrient supply of tumor (23).

Bevacizumab (24), a humanized monoclonal antibody, was approved by the Food and Drug Administration (FDA) as the first angiogenesis inhibitor for the treatment of advanced colorectal cancer in 2004, and for first-line treatment of advanced NSCLC in 2006. Bevacizumab can inhibit tumor cell proliferation by specifically binding to VEGF, blocking the bind of VEGF and endothelial cell surface receptors, suppressing tumor neovascularization and normalizing abnormal blood vessels (22). However, bevacizumab is not recommend for advanced lung squamous cell carcinoma due to its high risk of hemoptysis and fatal bleeding associated with squamous cell carcinoma (25).

Rh-endostatin is one of antitumor vascular targeting drugs with a wide range of targets that distinguishes it from other single-target or multi-target anti-angiogenesis drugs. Rh-endostatin can specifically target tumor vascular endothelial cells, promoting tumor vascular normalization and reducing hypoxia. In turn, it facilitates the infiltration of CD8+ T lymphocytes into tumors, decreasing the population of immunosuppressive myeloid-derived suppressor cells (MDSCs) and M2-like tumor-associated macrophages (TAMs), increasing the population of myeloid dendritic cell (mDC) and M1-like TAMs, ultimately improving the inhibitory state of tumor immune microenvironment (12, 15, 26, 27). As a result, rh-endostatin is considered one of the most promising medications in antitumor vascular treatment. In addition, rh-endostatin has the characteristics of endogenous, mild toxicity and low drug resistance.

There is an important relationship between tumor immune microenvironment and tumor angiogenesis. Angiogenesis inhibitors can not only eliminate the blood vessels required for tumor growth, but also regulate the tumor microenvironment and improve the efficacy of immunotherapy by promoting the proliferation and maturation of immune effector cells. At the same time, ICIs can enhance the efficacy of angiogenesis inhibitors by restoring the body’s normal immune response and promoting vascular changes (13–15). The combination of ICIs and anti-angiogenesis therapy can overcome ICIs resistance and improve the prognosis of patients, which is a new treatment model. Therefore, there may be synergistic effect between ICIs and angiogenesis inhibitors in antitumor therapy, providing a theoretical basis for their combined application.

In many studies, ICIs plus anti-angiogenesis therapy in patients with advanced NSCLC had shown good antitumor efficacy and a manageable safety profile. For example, IMpower 150 study (28) demonstrated that atezolizumab plus bevacizumab and chemotherapy could significantly prolong mPFS (8.3 months vs 6.8 months), mOS (19.2 months vs 14.7 months, HR = 0.78, *P* = 0.02) and ORR (63.5% vs 48.0%) in patients with advanced non-squamous NSCLC, compared with bevacizumab plus chemotherapy. Additionally, a Phase Ib trial (29) showed that the combination of anlotinib and sintilimab had good efficacy, durability, and tolerability in patients with advanced NSCLC with negative driver gene for first-line treatment, with ORR was 72.7% (95% CI 49.8%-89.3%), DCR was 100%(95% CI 84.6%-100%), mPFS was 15 months, and one-year PFS rate was 71.4% (95% CI 47.2%-86.0%). In a multicenter retrospective study (30), a total of 21 advanced NSCLC patients received camrelizumab combined with rh-endostatin and chemotherapy (76% for adenocarcinoma and 19% for squamous cell carcinoma), ORR and DCR were 71% and 100%, 1 patient received CR, and 11 patients (52%) were undergoing treatment, mPFS was not achieved, and the safety is good. Xu et al. (31) suggested that ICI plus anti-angiogenesis therapy may be beneficial to the subsequent treatment of patients with advanced or metastatic NSCLC, mPFS was 5.0 months (95% CI 3.179-6.821),mOS was 14.3 months (95%CI 8.912-19.659), ORR and DCR were 10.3% and 72.4%. Moreover, Huang et al. (32) found that compared with ICI monotherapy, ICI combined with anti-angiogenesis therapy as second-line or later treatment in advanced lung adenocarcinoma showed better survival prognosis, mPFS (5.1m vs 2.0m, HR = 0.551, 95% CI 0.337-0.902, *P* = 0.002) and mOS (14.3m vs 8.4m, HR = 0.549, 95% CI 0.305-0.990, *P* = 0.046). Lu et al. (33) showed that 34 patients with advanced NSCLC received nivolumab plus rh-endostatin, ORR was 41.2% (14/34, 95% CI 23.7%-58.6%), DCR was 64.7% (22/34, 95% CI 47.8%-81.6%), mPFS was 6.8 months (95% CI 1.1-12.1), the mOS was 17.1 months (95% CI 6.6-27.6), and the one-year survival rate was 64.4% (95% CI 46.2-82.6%).

The aforementioned data suggests that the combination of ICIs and angiogenesis inhibitors exists satisfactory antitumor efficacy and safety. Additionally, it has been found that ICIs can reduce the host immune tolerance to tumors, and enhance the abscopal effect of radiotherapy (RT) and further amplify the anti-tumor immune response (34). Meanwhile, RT can enhance the anti-tumor effect of the immune system by up-regulating immunogenic cell surface markers such as intercellular cell adhesion molecule 1 (ICAM-1), major histocompatibility complex class 1 (MHC-1) and Fas (35). RT can also facilitate T cell infiltration by altering the vascular phenotype and promoting the release of chemokines, providing a more favorable immune micro-environment for exerting an effective anti-tumor response (36). Therefore, ICIs and RT also have potential synergistic mechanism in anti-tumor therapy. A multi-center phase III randomized controlled trial, PACIFIC (37), evaluated Durvalumab in patients with stage III unresectable NSCLC after concurrent chemoradiotherapy. The results indicated that Durvalumab group significantly prolonged mPFS (16.8 months vs 5.6 months, P < 0.001), median time to death or distant metastasis (23.2 months vs 14.6 months, P < 0.001), and 5-year survival rate (42.9% vs 33.4%), and with similar safety profiles, compared with placebo group. Another randomized, double-blind, multicentre, phase 3 trial (38) included a total of 381 patients with locally advanced, unresectable, stage III NSCLC after concurrent or sequential chemoradiotherapy. The results show that PFS assessed was significantly longer with sugemalimab than with placebo (9.0 months vs 5.8 months, P = 0.003). Thus, the combination of ICIs and RT could be another one of the solutions to increase the fraction of responding patients to ICIs, which still needs to be further confirmed by more researches.

Therefore, in our pilot study, we found that ICIs plus anti-angiogenesis therapy as second-line treatment prolonged clinical survival in advanced NSCLC with negative driver gene, compared to ICIs monotherapy. In ICIs combination therapy group, mPFS and mOS were 8.3 months (95% CI 6.811-9.722) and 18.0 months (95% CI 12.143-23.923). In ICI monotherapy, mPFS and mOS were 3.7 months (95% CI 3.385-4.082) and 9.6 months (95% CI 4.733-14.533). In terms of safety, we did not observe a significant difference in the incidences of AEs between the two groups (P > 0.05), which is consistent with previous studies (9, 32). The majority of patients experienced grade 1-2 AEs, with no treatment-related deaths occurred. The vast majority of AEs can be managed with symptomatic treatment without affecting the continuation of the study, suggesting that ICI combination therapy was well tolerated.

However, this study has numerous drawbacks. First, the sample size of this study is limited, which may have an impact on the outcomes. Second, the treatment regimens in this study involved four different ICI, which may have confounding effects on efficacy.

In conclusion, our findings suggest that compared with ICIs monotherapy, the combination of ICI and anti-angiogenesis as second-line therapy in advanced NSCLC with negative driver gene gained a higher DCR, prolonged PFS and OS, with a manageable safety. Further randomized controlled trials with larger sample sizes are needed to confirm our findings.

## Data availability statement

The original contributions presented in the study are included in the article/supplementary material. Further inquiries can be directed to the corresponding author.

## Ethics statement

The studies involving humans were approved by Ethics Committee of the Third Affiliated Hospital of Anhui Medical University. The studies were conducted in accordance with the local legislation and institutional requirements. The participants provided their written informed consent to participate in this study.

## Author contributions

Concept and design: XS. Acquisition, analysis, or interpretation of data: BY. Drafting of the manuscript: BY. Critical revision of the manuscript: XS, YZL, JD, HY. Administrative, technical, or material support: XS, YZL. Supervision: XS. All authors contributed to the article and approved the submitted version.
